# High‐Performance Dopamine‐Based Supramolecular Bio‐Adhesives

**DOI:** 10.1002/marc.202400345

**Published:** 2024-06-06

**Authors:** Maximilian J. L. Hagemann, Lewis Chadwick, Marcus J. Drake, Darryl J. Hill, Benjamin C. Baker, Charl F. J. Faul

**Affiliations:** ^1^ School of Chemistry University of Bristol Bristol BS8 1TS UK; ^2^ Department of Surgery and Cancer Imperial College du Cane road London W12 0HS UK; ^3^ School of Cellular and Molecular Medicine University of Bristol Bristol BS8 1TD UK

**Keywords:** biocompatible adhesives, Supramolecular glue, surgical adhesives

## Abstract

The need for wound closure or surgical procedures has been commonly met by the application of sutures. Unfortunately, these are often invasive or subject to contamination. Alternative solutions are offered by surgical adhesives that can be applied and set without major disruption; a new class of supramolecular‐based adhesives provides potential solutions to some of these challenges. In this study, a series of polymers utilizing dopamine as a self‐assembling unit are synthesized. It is found that these motifs act as extremely effective adhesives, with control over the mechanical strength of the adhesion and materials’ tensile properties enabled by changing monomer feed ratios and levels of cross‐linking. These materials significantly outperform commercially available bio‐adhesives, showing yield strengths after adhesion at least two times higher than that of BioGlue and Tisseel, as well as the ability to re‐adhere with significant recovery of adhesion strength. Promisingly, the materials are shown to be non‐cytotoxic, with cell viability > 90%, and able to perform in aqueous environments without significant loss in strength. Finally, the removal of the materials, is possible using benign organic solvents such as ethanol. These properties all demonstrate the effectiveness of the materials as potential bio‐adhesives, with potential advantages for use in surgery.

## Graphical abstract

In this study, a series of carefully designed polymers, utilizing dopamine as a hydrogen‐bonding self‐assembling unit, are synthesized. It is found that these motifs act as extremely effective single‐component adhesives, with control over the strength of adhesion and materials’ tensile properties enabled by changing monomer feed ratios and levels of cross‐linking.

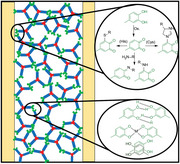

## 1 Introduction

The impact and need for surgery is an increasing issue worldwide, with the number of procedures increasing by over 33% in 8 years to a total of 313 million per annum (figures for 2012).^[^ ^1^ ^]^ With the total mortality rate of these procedures being nearly 8 million deaths per annum,^[^ ^2^ ^]^ more research is required to reduce the mortality rate significantly. With sepsis being the primary cause of post‐operative deaths,^[^ ^3^ ^]^ quick wound closure and shorter time spent in surgery are important goals to achieve.^[^ ^4^ ^]^

One way of reducing operation times,^[^ ^5^ ^]^ is the introduction of surgical adhesives,^[^ ^6^ ^]^ which could reduce problems associated with surgical sutures. Conventional sutures generally use a curved atraumatic needle, which is passed around the repair site in an arc through adjacent tissue with a needle holder. When the needle emerges on the far side of the repair site it is pulled through to bring the suture material into position, which is then secured by tying a knot. For repairs needed in deep locations, there are several potential disadvantages to using sutures. Space is confined, which means manipulating the needle into position and tying the knot is difficult and sometimes traumatic for the tissue. If the knot is not tied reliably there is a risk it could unravel and hence fail to achieve its required purpose. The arc followed by the needle might encroach on nearby structures, potentially leading to damage to blood vessels, nerves or adjacent organs (notably the ureter in abdominal surgery). The situation is particularly difficult in open surgery, due to difficulties with illumination and vision. The used glues exhibit further advantages in comparison with standard surgical procedures, e.g., they can be used in a wide variety of settings without the need for expert application, result in excellent cosmetic results, eliminate the need for suture removal (and added complications if such procedures are not performed by trained medical staff), and are useable in situations where mechanical fastening is unsuitable or very invasive.^[^ ^7^, ^8^ ^]^

Applications of surgical adhesives in broader areas beyond just surgical settings are further topics of current research.^[^ ^6^ ^]^ These areas include the effective closure of small oozing wounds,^[^ ^9^, ^10^, ^11^ ^]^ with the aim to provide short‐term solutions in situations where surgery might not be possible or appropriate.^[^ ^12^ ^]^ Further application areas for surgical adhesives are the fixation of bone fractures,^[^ ^13^ ^]^ nerve anastomosis,^[^ ^14^ ^]^ integration of biomaterial implants to cartilage^[^ ^15^ ^]^ and prosthetic mesh fixation,^[^ ^16^ ^]^ presenting these materials as interesting alternatives for or addition to many conventional surgical procedures.

However, significant challenges still exist for the wider area of bio‐adhesion, including strength of adhesion of synthetic solutions compared with biological counterparts. Current commercial solutions that are available on the market include BioGlue, with a yield strength (YS) of 30 kPa, and Tisseel with a YS of 7.6 kPa.^[^ ^17^ ^]^ These materials show significantly weaker YSs than exhibited by tendons (16.5 MPa), and even muscles (YSs of skeletal muscles: 350 kPa and cardiac muscle: 100 kPa), and are therefore still in need of further improvement.^[^ ^18^ ^]^ Moreover, leaching of bio‐glue components, e.g., in fibril‐based glues, can result in viral infections such as human immunodeficiency virus (HIV) and hepatitits;^[^ ^19^, ^20^ ^]^ these issues can limit their wider use beyond dermal applications.^[^ ^21^ ^]^ Additionally, undesired stiffness of the material can limit their application and make it unsuitable for applications where more tissue‐like behavior is necessary, e.g., in aortic root replacement.^[^ ^22^ ^]^ Ultimately, a range of options needs to be explored to meet the various demands of the potential clinical applications, especially since the rigorous regulatory requirements for human use mean that many proposed solutions will not be licensed, or may not be commercially viable.

Closely related to this established area of medical procedures (and the challenges faced) is the growing body of research into soft artificial actuators (or muscles), especially biocompatible actuators for future in vivo use.^[^ ^23^ ^]^ This interdisciplinary field, which includes contributions from materials science, chemical engineering, mechanical engineering, electrical engineering, and chemistry,^[^ ^24^ ^]^ is focused on the development of devices capable of reversible contraction, expansion, and rotation, imitating the movement of biological muscles.^[^ ^23^ ^]^ These actions can be induced by thermal,^[^ ^25^ ^]^ electrochemical,^[^ ^26^ ^]^ pneumatic,^[^ ^27^ ^]^ and light stimuli.^[^ ^28^ ^]^ The focus areas for the application of such biocompatible actuators are to provide solutions for, among others, incontinence^[^ ^29^ ^]^ and general sarcopenia (i.e., muscle loss due to increased age),^[^ ^30^ ^]^ thus contributing to increased quality of life. As the field moves into in vivo applications, adhesion of the artificial actuators to biological tissue, tendons or bones poses significant challenges for many of these applications, especially where strong anchoring of actuators is required for their operation.^[^ ^31^ ^]^

Supramolecular adhesives are a class of materials that demonstrate the necessary properties for this demanding and broad application area.^[^ ^32^ ^]^ In this field of research intramolecular interactions such as hydrogen bonding, π‐stacking, and metal coordination are utilized to enhance or introduce attractive properties (in addition to adhesion), including the ability to self‐heal.^[^ ^33^, ^34^ ^]^ Self‐healing can improve the longevity of these materials as already shown in different application areas, e.g., spacecraft construction,^[^ ^33^ ^]^ by self‐healing damages at the micro level and thus inhibiting slow degradation of the material over prolonged periods.

**Scheme 1:**
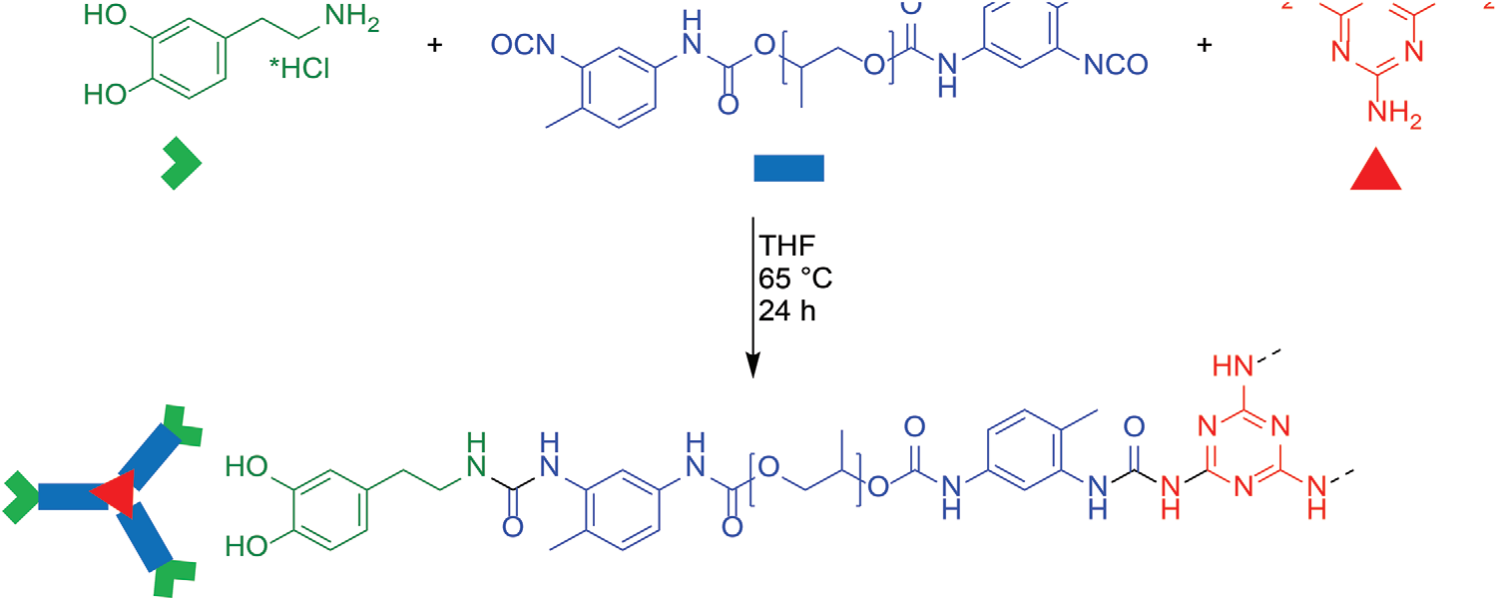
Reaction scheme for the synthesized target compounds.

Even if materials exhibit such attractive properties, they still leave the challenge of providing strong adhesion between synthetic and biological material in an aqueous environment (i.e., in bodily fluids). However, the field of mussel‐inspired adhesion provides a promising approach for an in vivo aqueous‐based setting.^[^ ^31^, ^35^, ^36^ ^]^ Adhesion in this case is achieved by catechol groups (**Scheme** **1**, green moiety), which introduces multiple supramolecular interactions, including intermolecular hydrogen bonding between hydroxyl groups,^[^ ^37^ ^]^ π‐stacking between benzyl moieties and metal coordination with the hydroxyl groups.^[^ ^38^, ^39^ ^]^ Additionally, multiple sources suggest oxidation of the catechol groups to form quinone groups,^[^ ^40^, ^41^, ^42^ ^]^ which possess the ability to form covalent bonds with a variety of different groups. Some of these reactive groups are commonly present in the body, e.g., amines from amino acids, and imidazole or thiol moieties present in histidine and cysteine residues, respectively. The creation of such additional covalent bonds can result in an even stronger adhesion to organic material.

In this study, we aim to address the challenges related to the development of a suitable surgical bio‐adhesive and show here our efforts to develop a biocompatible material with exceptional adhesion (e.g., high yield and tensile strength) and resilience (e.g., stability and self‐healing abilities). To achieve this aim, especially in the aqueous in vivo environment where such solutions are required, we propose a combination of supramolecular interaction motifs based on urea groups, and mussel‐inspired adhesion as found in catechols. Additionally, while utilizing a simple synthetic procedure with easy purification methods, we explore the influence of different degrees of cross‐linking on the materials’ properties and function. Our approach limits the possibility of leaching of any undesired materials, thus ensuring the long‐term biocompatibility of these bio‐adhesives.

## 2 Experimental Section

### 2.1 Materials

All starting materials were purchased from Merck and used without further purification.

### 2.2 Synthesis of polymers Lin1 to Mel_5.0_

Typical synthesis, **Mel_1.5_**: Dopamine hydrochloride (Dop, 82.2 mg, 0.43 mmol, 3.0 eq.), toluene diisocyanate‐capped poly(propylene glycol) (PPG, 2300 M_n_, 1000 mg, 0.43 mmol, 3.0 eq.), and melamine (Mel, 27.4 mg, 0.22 mmol, 1.5 eq.) were dissolved in THF (30 mL), and the mixture reacted under reflux for 24 h. After cooling to room temperature, 1 m HCl (50 mL) was added, the reaction mixture was filtered and the residue was washed with H_2_O (150 mL) to yield the product as a white gel.

FT‐IR: *ṽ* (cm^−1^) = 3344 (N‐H_urea_), 2869 (C‐H_alkane_), 1536 (C = N_melamine_), 1086 (C‐O_ether_), 816 (C = N_melamine_).

Please see **Table** **1** for further details of the synthesis of polymers **Lin1** – **Mel_5.0_**, with varying melamine content.

**Table 1:** Molar ratios of the different starting materials for the synthesis of Lin_1_ to Mel_5.0_ following the described procedure, with the mass of Mel/Dop/PPG (m_Mel/Dop/PPG_), the amount of substance of Mel/Dop/PPG (n_Mel/Dop/PPG_) and the molar equivalents given as m_Mel/Dop/PPG_.

	m_Mel_ [mg]	n_Mel_ [mmol]	Mel [eq.]	m_Dop_ [mg]	n_Dop_ [mmol]	Dop [eq.]	m_PPG_ [mg]	n_PPG_ [mmol]	PPG [eq.]	Yield [%]
Lin1	0.00	0.00	0.0	82.2	0.43	3.0	1000	0.43	3.0	93
Mel_1.0_	18.3	0.14	1.0	82.2	0.43	3.0	1000	0.43	3.0	97
Mel_1.5_	27.4	0.22	1.5	82.2	0.43	3.0	1000	0.43	3.0	91
Mel_2.0_	36.6	0.29	2.0	82.2	0.43	3.0	1000	0.43	3.0	94
Mel_2.5_	45.7	0.36	2.5	82.2	0.43	3.0	1000	0.43	3.0	96
Mel_3.0_	54.8	0.43	3.0	82.2	0.43	3.0	1000	0.43	3.0	93
Mel_3.5_	64.0	0.51	3.5	82.2	0.43	3.0	1000	0.43	3.0	92
Mel_4.0_	73.1	0.58	4.0	82.2	0.43	3.0	1000	0.43	3.0	94
Mel_4.5_	82.3	0.65	4.5	82.2	0.43	3.0	1000	0.43	3.0	92
Mel_5.0_	91.4	0.70	5.0	82.2	0.43	3.0	1000	0.43	3.0	90

### 2.3 Characterization

Details of all characterization instrumentation and methods, as well as recorded spectra of all samples are provided in the Supporting Information.

## 3 Results and Discussion

To design a suitable class of materials that would provide all the required properties and functions as set out above, the following approach was followed: a) dopamine, Dop, was selected as the component to provide the required adhesive interactions for the intended application; b) poly(propylene glycol), PPG, was chosen as the polymeric component to provide combination of biocompatibility,^[^ ^43^, ^44^ ^]^ insolubility in aqueous media^[^ ^45^ ^]^ and a sufficient degree of flexibility; c) melamine, Mel, was used to introduce branching of the resulting adhesive polymers, thus increasing potential entanglement while also improving the intramolecular interactions between the polymers.^[^ ^46^ ^]^

In addition to a linear dopamine‐PPG control polymer (**Lin1**, no melamine), we synthesized nine systems (**Mel_1.0_** to **Mel_4._**, Table 1) with varying ratios of melamine as crosslinker (Scheme 1, Dop in green, PPG in blue, and Mel in red). It is noteworthy that all polymers were synthesized without the need for inert atmosphere reaction conditions or dry solvents.

**Figure 1:**
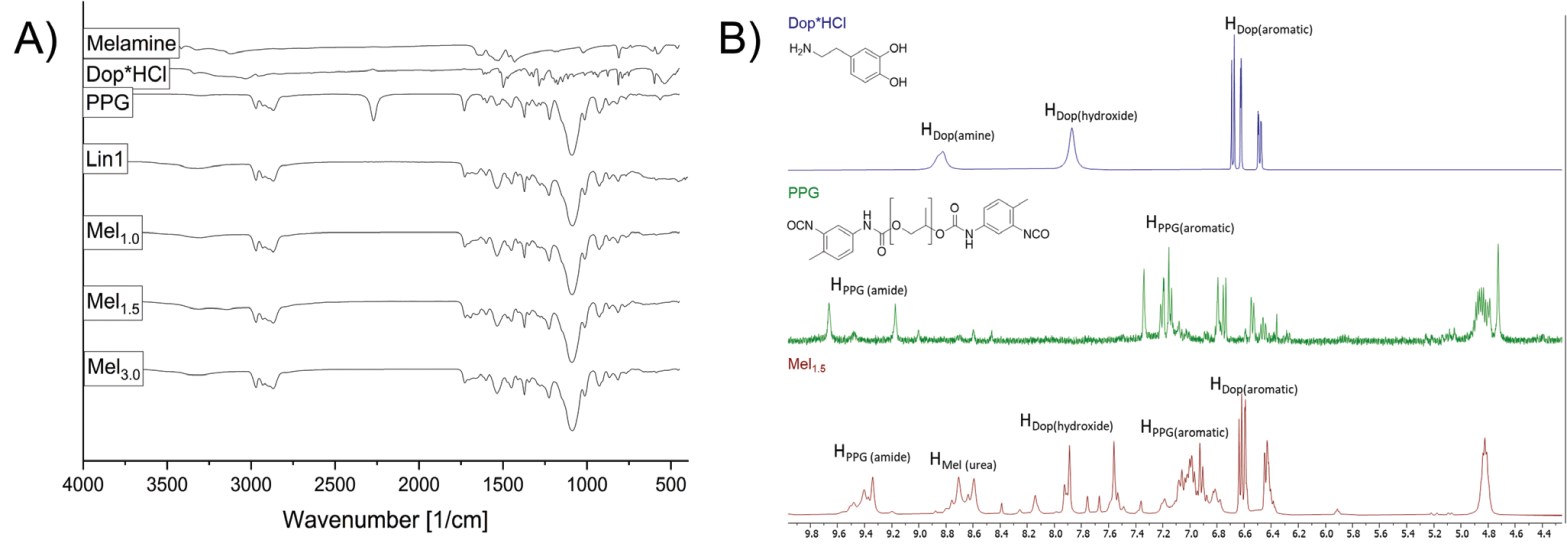
A) FTIR spectra of starting materials, linear control polymer **Lin1**, and selected polymers **Mel_1.0_**, **Mel_1.5_**, and **Mel_3.0_**; B) NMR spectra of starting materials Dop and PPG, and polymer **Mel_1.5_** as representative examples.

FTIR analyses confirmed the successful formation of the product by the disappearance of the isocyanate (N═C═O─ stretching) signal at 2272 cm^−1^ in comparison with the starting material PPG (**Figure** **1**A, highlighted Area II). This successful addition can be further verified by the stronger absorbance of the typical N‐H stretching vibration at 3344 cm^‐1^ in accordance with the formation of urea groups (Figure 1A, highlighted Area I) for all samples. The formation of urea groups is further supported by the widening of the C═O typical signals (ca. 1700 cm^−1^, Figure 1A, highlighted Area III). The successful incorporation of the melamine core is confirmed by the new signal at 1539 cm^−1^ (Figure 1A, highlighted Area IV, **Mel_1.0_ Mel_1.5_** and **Mel_3.0_**), which is assigned to the C═N stretching of the melamine core and is also seen in the FTIR spectrum of the starting material. See FTIR analyses for all samples in Figure S4 (Supporting Information).

To further confirm the successful formation of the desired products, ^1^H NMR spectra were recorded for all products (see Figure S6, Supporting Information) in DMSO‐*d_6_*. It is worth noting that all spectra show significant integrals for the repeating propylene glycol unit, which averages 32 repeating units per PPG chain. This significant difference in peak intensity of propylene glycol signal in comparison to that of the end groups resulted in challenges to evaluate the obtained data (see, for example, the initial ^1^H NMR spectrum of **M_1.5_** in Figure S5, Supporting Information). Increasing the number of measurements per spectrum to 256 (from the typical 8 measurements), yielded acceptable S/N ratios to enable end‐group analysis. Successful formation of the product was indicated by the shift of the aromatic protons of the aromatic PPG protons (H_PPG(aromatic)_, 6.9–7.1 ppm, Figure 1B) with the attached isocyanate group upon conversion to urea groups. Further proof of a successful reaction was the disappearance of the wide amine peak of the dopamine at 7.8 ppm (H_Dop(amine)_, Figure 1B). Furthermore, the appearance of new signals between 8.5 and 9.0 ppm, typical for the protons from urea moieties, underlines the successful formation of urea groups and therefore the formation of the desired product.^[^ ^47^ ^]^

### 3.1 Tensile Strength

**Figure 2:**
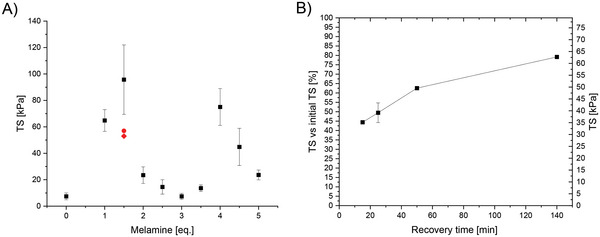
A) Average TS of **Lin1**‐**Mel_5_**_._ **_0_**; with **Mel_1.5_** a (ex situ and stored fully submerged in a PBS solution at room temperature for 1 week) and b (adhesive were dipped in PBS prior to sample preparation and left submerged in PBS for 1 week after contact) marked by a red circle and a red diamond, respectively. B) recovery properties of **Mel_1.5_** with a standard deviation of 4% for the measurement after 25 min (to test repeatability).

To test tensile strength of the synthesized polymers, materials were applied on an acrylic sheet and subsequently covered with a second sheet with a fixed, recorded overlap of ca. 13 mm (total overlap surface area of 3 cm^2^). The acrylic sheets were held together for 48 hours followed by tensile measurements. All mechanical tests were repeated 4 times to achieve the average yield strengths as shown in **Figure** **2**A.

The following trends were observed when samples with varying ratios of melamine were considered: a strong initial increase in the yield strength is evident, up to sample **Mel_1.5_** (with 1.5 eq. of melamine), averaging 96 kPa. Beyond this ratio, a dramatic decrease in tensile strength is observed, up to **Mel_3.0_**, with 3.0 eq. of melamine. A sudden increase is then observed again to 71 kPa for **Mel_4.0_**, however not reaching the adhesive properties of **Mel_1.5_**. Finally, tensile strength values decrease again for the final samples. From these investigations, the performance of **Mel_1.5_** (with a TS of 96 kPa) shows that the system with a slight excess of melamine is the most promising ratio to achieve TS values that easily and significantly outperform the commercially available glues, BioGlue (34 kPa)^[^ ^48^ ^]^ and Tisseel (7.6 kPa).^[^ ^17^ ^]^

To further explore the practical applicability of our materials, polymer **Mel_1.5′_** s ability for self‐healing and reusability was explored. After measuring the initial TSs following the above‐described procedure, the two sheets were completely separated and held apart for 60 seconds. The two separated slides were then held together for a specific period of time (the “recovery time” as shown on the x‐axis, Figure 2B) before the tensile strength tests were repeated. These measurements show a clear trend of recovery, with 80% recovery of the original TS values obtained after a recovery time of 140 min.

A further advantage of our supramolecular adhesive approach is the ease of application of the material. In comparison with other adhesives that require in situ polymerization of two components,^[^ ^39^, ^49^, ^50^ ^]^ our materials can be directly applied to a biological matrix of interest without the need for any (polymerization) reaction. Additionally, exposure of a test sample to ethanol leads to total loss of adhesion within 40 s (see Video S1, Supporting Information), and would therefore allow for the removal of the supramolecular adhesive after use (e.g., post‐surgery recovery).

With these promising results, we continued to explore the potential of **Mel_1.5_** for use as a surgical adhesive. The adhesive performance was therefore investigated in phosphate‐buffered saline (PBS) to mimic in vivo conditions. For this purpose, two different approaches were chosen: in the first approach, the samples (two acrylic sheets connected by the adhesive, **Mel_1.5_** a, red circle in Figure 2A) were prepared ex situ and stored fully submerged in a PBS solution at room temperature for 1 week prior to mechanical testing. In the second approach (**Mel_1.5_** b, red diamond in Figure 2A), the sheets and adhesive were dipped in PBS prior to sample preparation and left submerged in PBS for 1 week after contact. YSs of 57 and 53 kPa, respectively, were achieved under these conditions (see Figures S12, Supporting Information). These values are still higher than the above‐mentioned commercial adhesives, with the potential for further increases after optimization.

After exploring the impressive adhesive properties of our materials, we employed dilution NMR experiments to gain an understanding of the involved inter‐ and intramolecular supramolecular interactions. For the dilution ^1^H NMR study, an initial concentration of 0.8 g mL^−1^ was chosen and diluted 50% with every dilution step. The solution was diluted 4 times resulting in concentrations of 0.8, 0.4, 0.2, and 0.1 g mL^−1^, respectively. NMR spectra were recorded at each concentration. With decreasing concentration, peaks of the OH_Dopamine_ and of all urea groups shifted to higher field and sharpened (see Figures S7–S10, Supporting Information). This sharpening of peaks can be explained by the absence of delocalization of protons due to hydrogen bonding, and thus is a sign of hydrogen bonding in the DMSO‐d_6_ solution. The observed shifts are typical for deshielding effects caused by π‐stacking between aromatic groups.^[^ ^51^ ^]^ These data support the hypothesis of multiple intramolecular interactions between the molecules being present even in the solution state, suggesting this as the modality of self‐adhesion and surface adhesion.

To further verify the importance of the dopamine groups to the adhesive properties, four further versions of the linear polymer (**Lin1**) with different end groups were synthesized and analyzed. The groups tested contained a phenyl ring (**Lin2**), a phenol group (**Lin3**), a single alcohol (**Lin4**), and an ionic phenyl (**Lin5**) as end groups, respectively (see Table S2, Supporting Information). Insufficient adhesion was observed to conduct tensile testing experiments for these materials, further underlining the importance of the dopamine end groups.

### 3.2 Toxicity

Concerns regarding the cytotoxicity of these materials, at first glance, are moderate, as all materials used in this study have been or are being used in food, consumer products^[^ ^52^ ^]^ or medical devices. In addition, dopamine, where occurring naturally, even shows positive effects against multiple illnesses.^[^ ^53^ ^]^ In addition, our synthetic approach provides an opportunity to purify the adhesive material prior to application, thus avoiding any potential harmful leaching of starting materials into the biological environment. However, to provide definitive proof of the benign nature and to clarify the suitability of **Mel_1.5_** as a surgical adhesive, cell toxicity was investigated.

**Figure 3:**
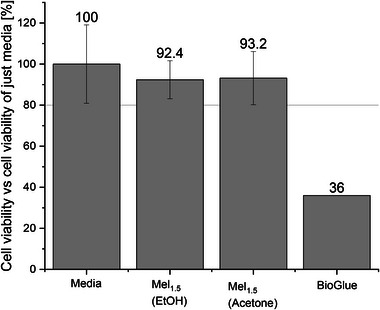
Cell viability test of non‐treated samples (Reference), ethanol, and acetone cast Mel_1.5_ samples (40 mg in each well seeded with 10^4^ HepG2 cells) and BioGlue.^[^ ^55^ ^]^ The line at 80% represents the limit for non‐toxic materials following the guidelines of the ISO10993‐5:2009 standard. Error bars were calculated from five repeat tests.

A standard Alamar Blue assay was carried out following a literature procedure.^[^ ^54^, ^55^ ^]^ For the preparation of the samples (**Mel_1.5_**) two different batches of **Mel_1.5_** were prepared at 200 mg mL^−1^ in ethanol and acetone, respectively. A volume of 200 µl of cell culture solution was seeded into a flat bottom 96‐well microplate at 1×10^4^ cells/well (HepG2 cells). The plate was incubated for 24 h, then the media in the microplates discarded and 200 µL of the **Mel_1.5_** solutions added to the cells. The cells were then incubated for another 24 h at 37 °C. Five replicates of each treatment solution were added to the cells. Alamar Blue as an indicator was used, acting as an oxidation–reduction sensitive indicator that changes fluorescence by reduction from the metabolism of living HepG2 cells. By comparing the fluorescence intensities (λ_excitation_ = 530 nm, λ_emission_ = 590 nm) of the controls (just containing the media) against the **Mel_1.5_**‐coated wells after an incubation time of 24 h, the amount of living cells can be determined. The two **Mel_1.5_** casting methods show cell viability of 92% and 93%, respectively, as seen in **Figure** **3**. Following the evaluation guidelines of the ISO10993‐5:2009 standard,^[^ ^56^, ^57^ ^]^ these values confirm that **Mel_1.5_** is non‐cytotoxic (as cell viability is higher than 80%) and therefore suitable for biological applications. It is noteworthy that the commercial product BioGlue only showed 36% cell viability, as reported by Murdock et al. using an MTT assay (see Figure 3 for comparison).^[^ ^55^ ^]^A standard Alamar Blue assay was carried out following a literature procedure.^[^ ^54^, ^55^ ^]^ For the preparation of the samples (**Mel_1.5_**) two different batches of **Mel_1.5_** were prepared at 0.2 mg mL^−1^ in ethanol and acetone, respectively. Alamar Blue acts as an oxidation—reduction sensitive indicator that changes fluorescence by reduction from the metabolism of living HepG2 cells. By comparing the fluorescence intensities (λ_excitation_ = 530 nm, λ_emission_ = 590 nm) of the controls (just containing the media) against the **Mel_1.5_**‐coated wells after an incubation time of 24 h, the amount of living cells can be determined. The two **Mel_1.5_** casting methods show cell viability of 92% and 93%, respectively, as seen in Figure 3. Following the evaluation guidelines of the ISO10993‐5:2009 standard,^[^ ^56^, ^57^ ^]^ these values confirm that **Mel_1.5_** is non‐cytotoxic (as cell viability is higher than 80%) and therefore suitable for biological applications. It is noteworthy that the commercial product BioGlue only showed 36% cell viability, as reported by Murdock et al. using an MTT assay (see Figure 3 for comparison).^[^ ^55^ ^]^

## 4 Conclusion

Successful development of this type of technology will enable surgeons to innovate and improve current operative approaches. For example, surgery to treat stress urinary incontinence in women sometimes uses a ribbon of connective tissue placed around the urethra. Currently, the operation secures the ribbon to the periosteum of the pubic bone by suturing. A significant incision is needed to visualize the area and secure the knots. Discomfort results from suturing into periosteum, and sometimes bone infection can result. Tissue glue as an alternative could be placed with a laparoscopic instrument, enabling a much smaller incision. The pain and infection risk associated with suturing through the periosteum would therefore be avoided.

Additionally to these advantages, we show easy removability of our surgical adhesives, e.g. after sufficient healing time, by applying simple and benign organic solvents such as ethanol. Through the integration of supramolecular moieties, we were able to show recovery rates to 80% of the original strengths of the surgical adhesives within just 140 min. This observation of 80% recovery of the original TS values after a recovery time is important, as physical exertion by the patient is likely to affect the adhesion sites in many potential contexts of use. When used in vivo, physical challenges imposed on the adhesive will not be as severe as the in vitro testing protocol described above.

To further test the applicability of the material in biological applications the stability in PBS and cytotoxicity of adhesive **Mel_1.5_** was investigated. Both investigations showed promising first results, with the Alamar blue tests showing no cytotoxicity of the material and the PBS study just showing a small decrease of the adhesion (still being stronger than comparable adhesives on the market).

For future research, some challenges still need to be addressed. First, the improvement of the bio‐adhesion by the addition of other compounds with more hydrogen‐bonding moieties should be investigated. Owing to the synthetic procedures presented, the adhesive and mechanical properties of our materials have the potential to be further tuned and optimized in a facile fashion.

Owing to the solubility and processability of our adhesives, easier processibility of the material via 3D‐printing could be investigated. 3D printing could be used as a tool for easier preparation and implementation of bionic composites to ensure adhesion of soft actuators as implantable muscles. Further potential applications can be explored for the production of bespoke and tailor‐made adhesive patches for fast and facile application and integration during surgical procedures. Ease of application is exceptionally important in the surgical context. Potentially, tissue adhesion should enable rapid and reliable repair by placement of the adhesive directly into the repair site. Well‐designed approaches to the delivery of the adhesive, supported with light source and visualization, will support effective repair achieved with less dissection than is currently required for suture placement. Such developments will also avoid the risk of damage to adjacent structures. The ability of the adhesive to perform in the range of environments demonstrated supports its potential use in several parts of an operation or surgical procedure. This could include securing the operation site, repair of unintended tissue damage, and minimally traumatic fixation of implants. There is also the possibility to achieve very precise repairs, by dissolving in ethanol to deliver the adhesive through a tube small enough to fit along the instrument channel of a laparoscope, and hence inject it with direct visualization by a laparoscope.

Overall, the ability to exploit supramolecular binding motifs for bio‐adhesive provides a strong foundation for the further improvement and application of surgical adhesives in a wide variety of clinical and soft robotics application areas.

## Conflict of Interest

The authors declare no conflict of interest.

## Supporting information

Supporting Information

## Data Availability

The data that support the findings of this study are available in the supplementary material of this article.
