# Supplementary material for: High‐Performance Dopamine‐Based Supramolecular Bio‐Adhesives
Source: Macromol Rapid Commun. 2024 Jun 6;45(23):2400345. doi: 10.1002/marc.202400345 (PMC11628360; doi:10.1002/marc.202400345)
Supplement: Supplementary file 1 — Supporting Information [file MARC-45-2400345-s001.docx]

High-Performance Dopamine-Based Supramolecular Bio-Adhesives

Maximilian J. L. Hagemann,^1^ Lewis Chadwick,^1^ Marcus J. Drake,^2^ Darryl J. Hill,^3^ Benjamin C. Baker,^1^* and Charl F. J. Faul^1^*

1 School of Chemistry, University of Bristol, Bristol BS8 1TS, UK

2 Department of Surgery and Cancer, Imperial College, du Cane road, London, W12 0HS, UK

3 School of Cellular and Molecular Medicine, University of Bristol, Bristol BS8 1TD, UK

[ben.c.baker@bristol.ac.uk](mailto:ben.c.baker@bristol.ac.uk); [charl.faul@bristol.ac.uk](mailto:charl.faul@bristol.ac.uk)

Supramolecular glue, surgical adhesives, biocompatible adhesives

**Contents**

[1. Synthesis 2](#_Toc159443072)

[2. Analytical methods 2](#_Toc159443073)

[3. Analysis 5](#_Toc159443074)

[3.1. FT-IR spectroscopy 6](#_Toc159443075)

[3.1.1. Spectra 6](#_Toc159443076)

[3.2. NMR spectrometry 6](#_Toc159443077)

[3.2.2. Spectra 7](#_Toc159443078)

[3.2.3. Dilution NMR 8](#_Toc159443079)

[4. Tensile-Testing 10](#_Toc159443080)

[5. Removability 11](#_Toc159443081)

# **Synthesis**

Dopamine hydrochloride (**Dop**, 82.2 mg, 0.43 mmol, 3.0 eq.), toluene diisocyanate-capped poly propylene glycol (**PPG**, 1.00 g, 2300 M_n_, 0.43 mmol, 3.0 eq.) and melamine (**Mel**, from 1 – 5.0 eq. Table S1) were dissolved in THF (30 mL), and the mixture was refluxed for 24 h. After cooling to room temperature, the solvent was removed under reduced pressure and the residue was washed with H_2_O (150 mL) to yield the product as a white gel.

**Table S1:** Amounts used for the synthesis of **Lin1** to **Mel_5.0_** following the above described procedure, with the mass of **Mel**/**Dop**/**PPG** (m**_Mel_**_/_**_Dop_**_/_**_PPG_**), the amount of substance of **Mel**/**Dop**/**PPG** (n**_Mel_**_/_**_Dop_**_/_**_PPG_**_)_ and the molar equivalents given as **Mel_m_**/**Dop_m_**/**PPG_m_**.

|  | m_Mel_ [mg] | n_Mel_ [mmol] | Mel_m_ [eq.] | m_Dop_  [mg] | n_Dop_ [mmol] | Dop_m_  [eq.] | m_PPG_ [mg] | n_PPG_ [mmol] | PPG_m_ [eq.] | Yield  [%] |
| --- | --- | --- | --- | --- | --- | --- | --- | --- | --- | --- |
| **Lin1** | 0.00 | 0.00 | **0.0** | 82.2 | 0.43 | **3.0** | 1000 | 0.43 | **3.0** | 93 |
| **Mel_1.0_** | 18.3 | 0.14 | **1.0** | 82.2 | 0.43 | **3.0** | 1000 | 0.43 | **3.0** | 97 |
| **Mel_1.5_** | 27.4 | 0.22 | **1.5** | 82.2 | 0.43 | **3.0** | 1000 | 0.43 | **3.0** | 91 |
| **Mel_2.0_** | 36.6 | 0.29 | **2.0** | 82.2 | 0.43 | **3.0** | 1000 | 0.43 | **3.0** | 94 |
| **Mel_2.5_** | 45.7 | 0.36 | **2.5** | 82.2 | 0.43 | **3.0** | 1000 | 0.43 | **3.0** | 96 |
| **Mel_3.0_** | 54.8 | 0.43 | **3.0** | 82.2 | 0.43 | **3.0** | 1000 | 0.43 | **3.0** | 93 |
| **Mel_3.5_** | 64.0 | 0.51 | **3.5** | 82.2 | 0.43 | **3.0** | 1000 | 0.43 | **3.0** | 92 |
| **Mel_4.0_** | 73.1 | 0.58 | **4.0** | 82.2 | 0.43 | **3.0** | 1000 | 0.43 | **3.0** | 94 |
| **Mel_4.5_** | 82.3 | 0.65 | **4.5** | 82.2 | 0.43 | **3.0** | 1000 | 0.43 | **3.0** | 92 |
| **Mel_5.0_** | 91.4 | 0.70 | **5.0** | 82.2 | 0.43 | **3.0** | 1000 | 0.43 | **3.0** | 90 |

**Table S2**: Amounts of amines used to obtain the polymers **Lin1** to **Lin4**.

|  | Amine X | m [mg] | n [mmol] | Eq. |
| --- | --- | --- | --- | --- |
| **Lin1** | Dopamine hydrochloride | 127 | 0.86 | **2.0** |
| **Lin2** | Phenyl-ethylamine | 101 | 0.86 | **2.0** |
| **Lin3** | 4-Amino-phenol | 50.7 | 0.86 | **2.0** |
| **Lin4** | Ethanolamine | 90.6 | 0.86 | **2.0** |

# Analytical methods

FTIR spectroscopy

Fourier-transform infrared spectroscopy (FTIR) spectra were recorded on a PerkinElmer Spectrum 100 spectrometer with a universal ATR two modular accessory with a diamond crystal. The samples were in a solid state. Sample spectra were recorded at wavenumbers between 450–4000 cm^-1^ at a resolution of 4 cm^-1^ with 10 scans.

NMR spectrometry

Nuclear magnetic resonance spectra were recorded using a 400 MHz Jeol Jastec spectrometer. Sample resonances were referenced to the residual solvent. Due to the poor solubility of products caused by the high molecular weights of the products, deviations of some integrals are present.

Tensile Strength Testing

For the lap shear strength tests ASTM D3163 procedure were followed. Two plastic sheets with the dimensions 25.0 × 100 mm were connected using the synthesised adhesives. To achieve that 0.2 g of material were partially dissolved in methanol (for easier application) and applied on a roughly 3 cm^2^ large area on the tip of a plastic sheet (Figure S1). The substrates were allowed to dry on air between the two sheets for 24 h.


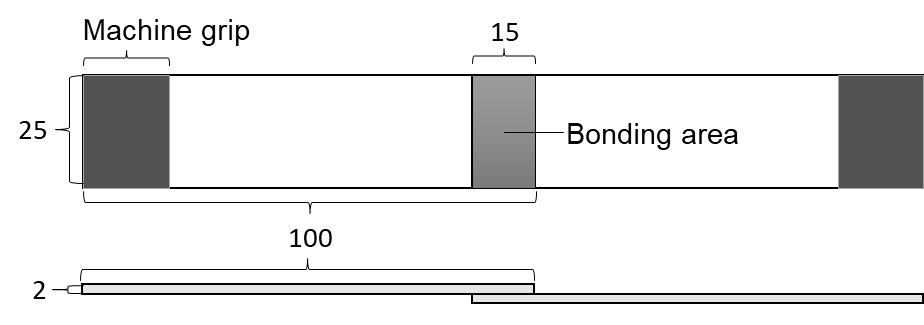


Bonding area

Machine grip

15

100

25

2

**Figure S1:** Schematic of the sample preparation of the lap shear test all values are in mm.

The test specimen were placed in the grips of a universal testing machine (Shimadzu AGS-X) and pulled at 1.0 mm/min until significant decrease of adhesion. Data was analysed using TrapeziumX and exported as excel data for further analysis.


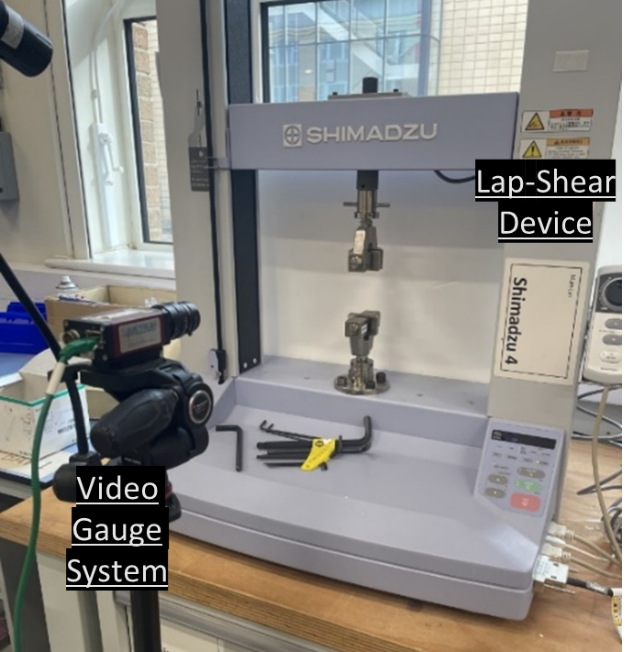


**Figure S2**: Setup-up of the Lap-Shear tests.

Biocompatibility screening

A 96-well plate was used to house the samples. Every grid of the well plate was treated with 0.1 mL of either solution of Mel_1.5_ in acetone or ethanol and the same amount of water were added to form films inside the wells and the solvents removed in vacuo. To this the cast material HepG2, human hepatoma cells in Dubeccos Phosphate Buffer Solution (DPBS), were added and incubated for 24 hours at 37 °C. 10% Alamar Blue in DPBS solution was then added and incubated for a further 24 hours.

UV/vis spectroscopy

UV/vis spectroscopy measurements were carried out using a Shimadzu UV-2600 spectrometer fitted with an ISR-2600 integrating sphere attachment.

Tensile-Strength Testing

For the adhesion measurements of all samples, an area of approximately 3.0 cm^2^ was covered with the polymer and the exact area was recorded. A force perpendicular to the plane was applied to the sample. Then strength of the adhesive was then determined by measuring the force required to shear the two plates apart.^13^ The main applied stress recorded during this lap shear test is the so-called shear stress.^14^

During the measurement the displacement of the plates was measured against the force exerted by the machine. The resulting graph is shown in Figure 9.

First the strain of the material was obtained using Equation (1), with ΔL being the change in length during the strain test and L being the original length of the material and is given as a unitless variable.

$$strain= \frac{\Delta L}{L}$$

(1)

Following that, the stress “σ” of the material was determined using Equation (2), with the stress being the quotient of the force created by the machine against the area of the adhesive.

$$stress \sigma= \frac{force}{area}$$

(2)

To further understand the mechanical properties of material **Lin1** a stress strain curve was created.


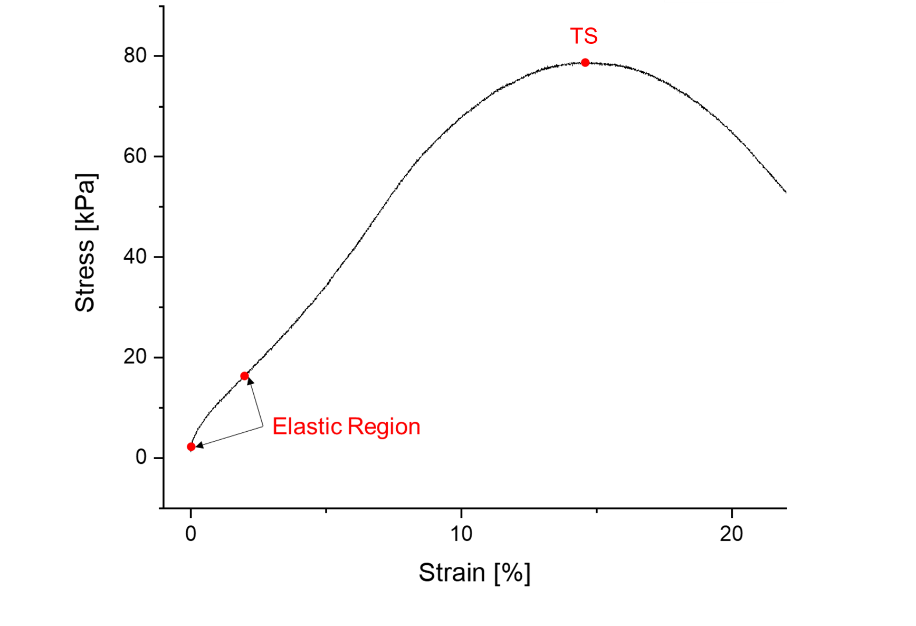


**Figure S3**: Stress-strain curve for **Mel_1.5_**

Analysing Figure 8 a variety of different constants can be obtained:

Tensile strength (**TS**)

Tensile strength or also ultimate tensile strength describes the maximum stress that a material can withstand prior breaking while withstanding an external force.

Yield strength (**YS**)

The yield strength describes the maximal stress that the material can withstand without being permanently deformed. Hereby, a linear relationship of stress and strain is observed up to this point. It is an important constant for adhesives, owing to a decrease of mechanical properties after plastic deformation.

Young’s Modulus (**YM**)

Young’s modulus (or elastic modulus) describes the stiffness of a material under load. It is determined in the from the elastic area of a material, where the material can be described by Hooke’s law. Hooke’s law states the direct proportionality between stress and strain of a material. To extract this data, the slope of the elastic area of the stress strain curve was taken (Equation (4)).

$$YM=\frac{\Delta\sigma}{\Delta strain}$$

(3)

# **Analysis**

## **FT-IR spectroscopy**

### **Spectra**

**Figure S4:** FT-IR spectra of all branched polymers **Mel_1.0_** to **Mel_5.0_**.

### **NMR spectrometry**

- - 1. **Examples**


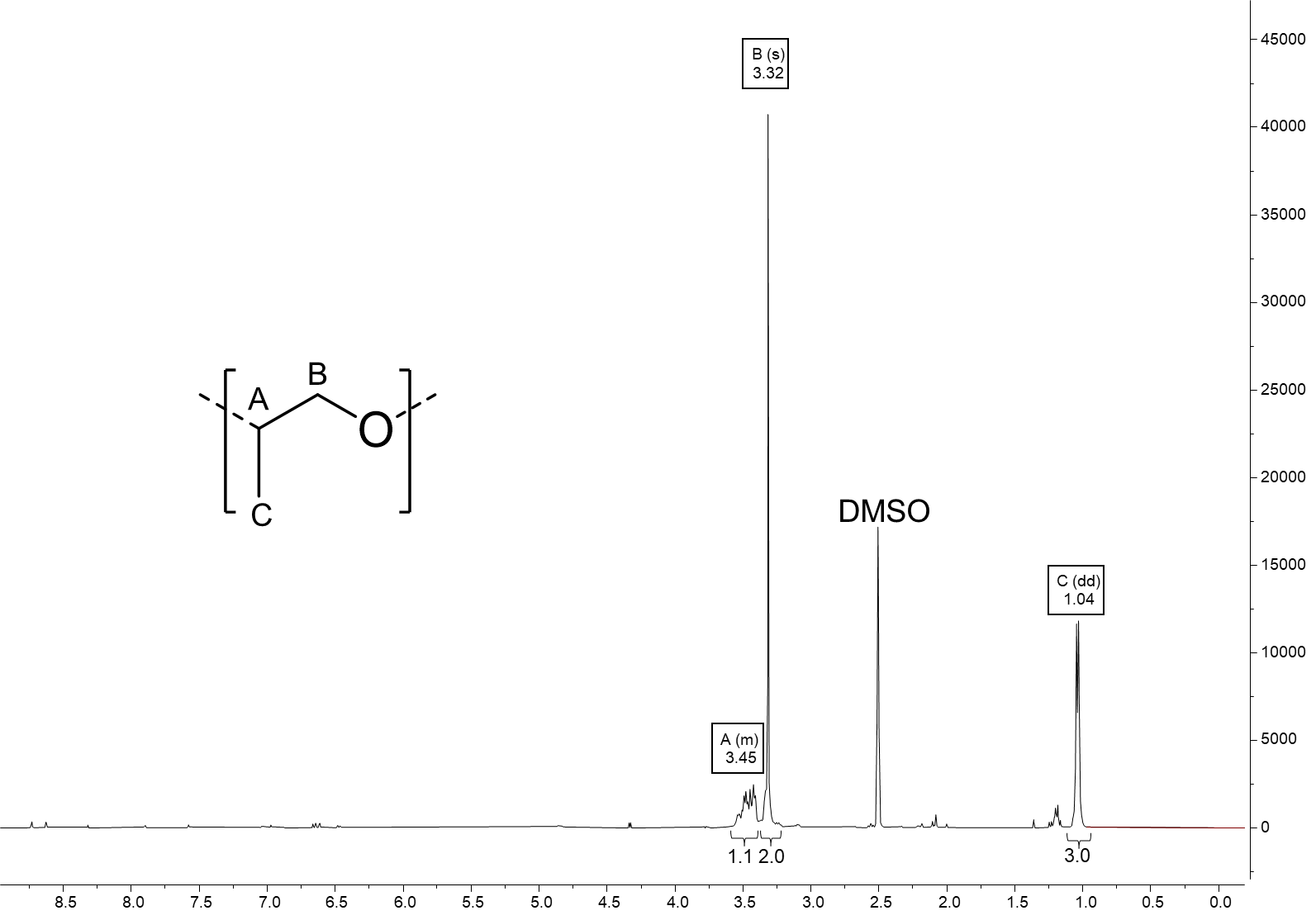


**Figure S5:** ^1^H-NMR spectrum of **Mel_2.0_**, assigning the peaks of the repeating PPG unit

### **Spectra**


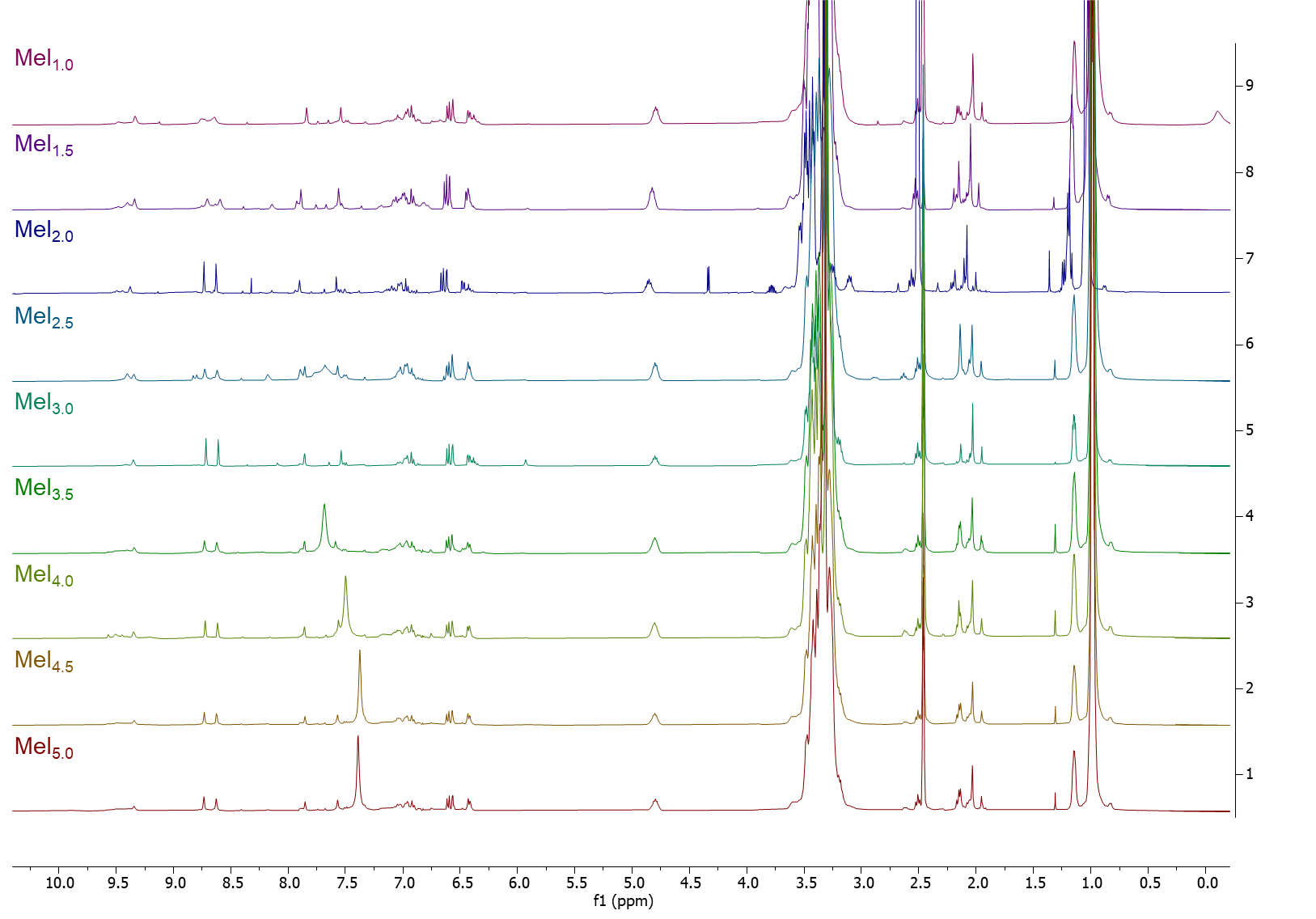


**Figure S6:** ^1^H-NMR of **Mel_1.0_** to **Mel_5.0_** in DMSO-d_6._

### **Dilution NMR**

For the dilution NMR study an initial concentration of 0.8 g/mL was chosen and diluted by 50% with every dilution step. The solution was diluted 4 times resulting in the concentrations of 0.8, 0.4, 0.2 and 0.1 g/mL, respectively. The spectra are being presented combined with the highest concentration at the top with decreasing concentrations towards the bottom. This study was used to show the intramolecular interactions of different moieties.


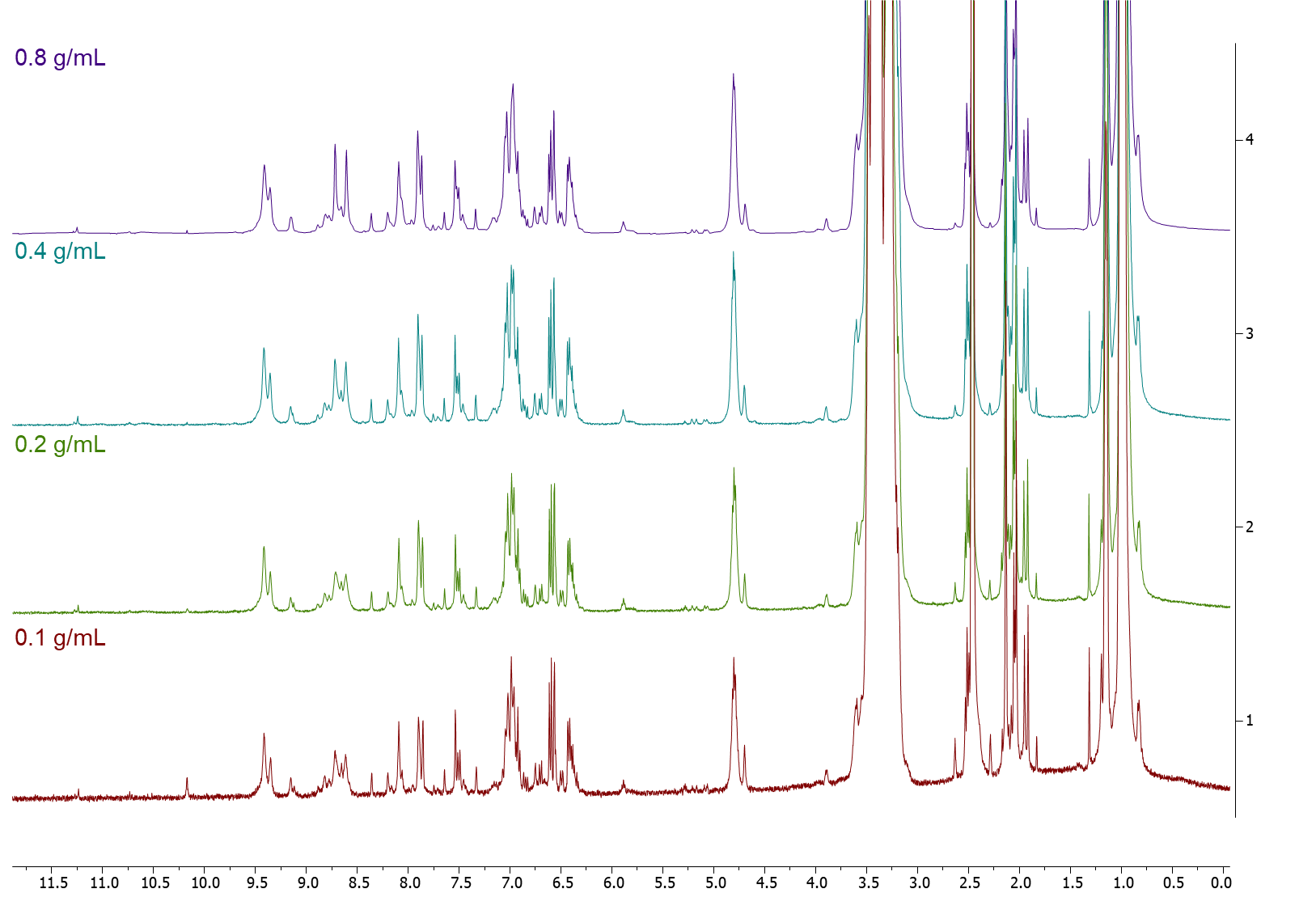


**Figure S7:** Spectra of the dilution measurements (0.8, 0.4, 0.2 and 0.1 g/mL from top to bottom, respectively).


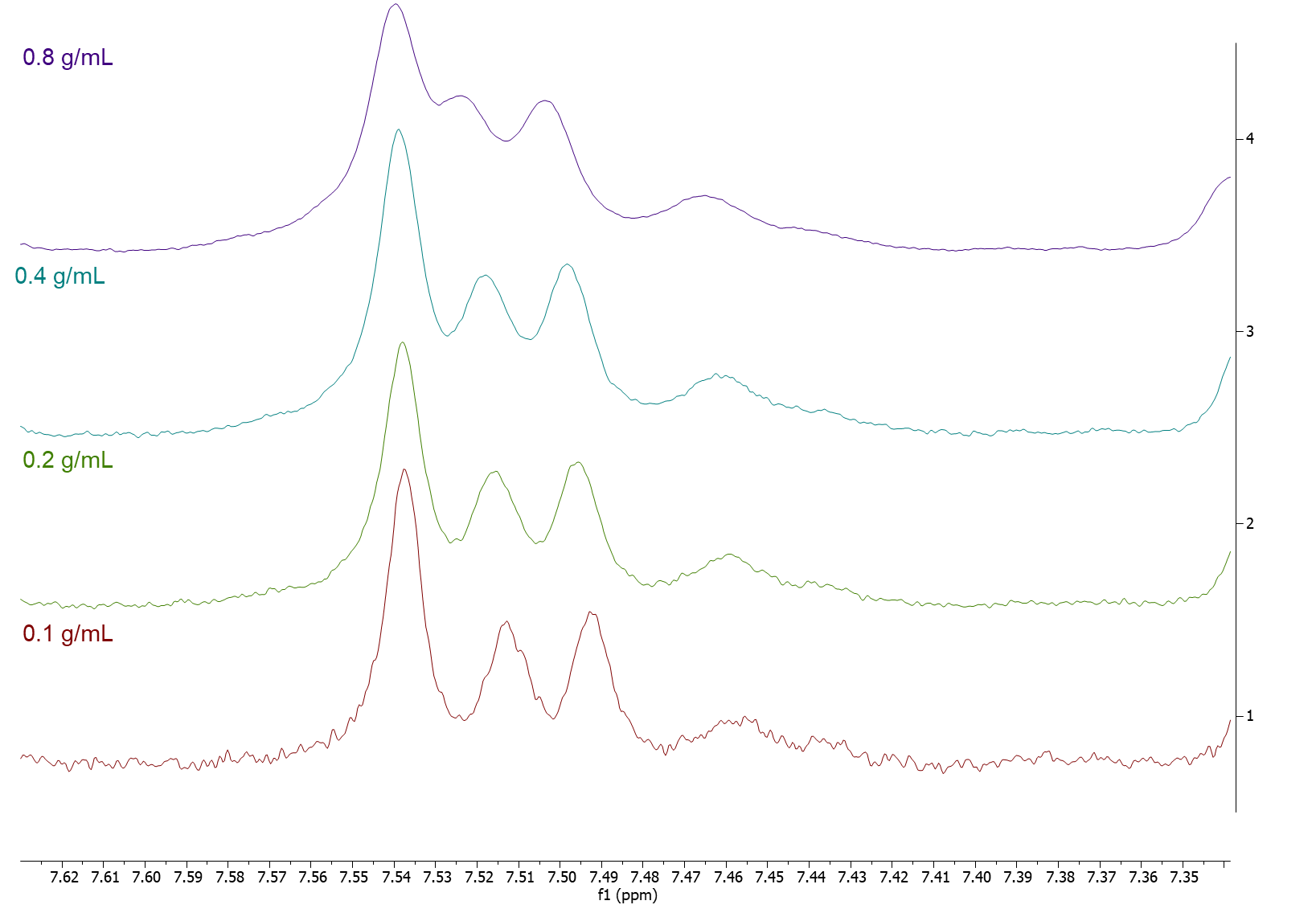


**Figure S8:** Zoomed-in version of the amide moiety of **Figure S7**, showing peak widening and a deep-field shift with decreasing degree of concentration, indicating a decrease of intramolecular interactions.


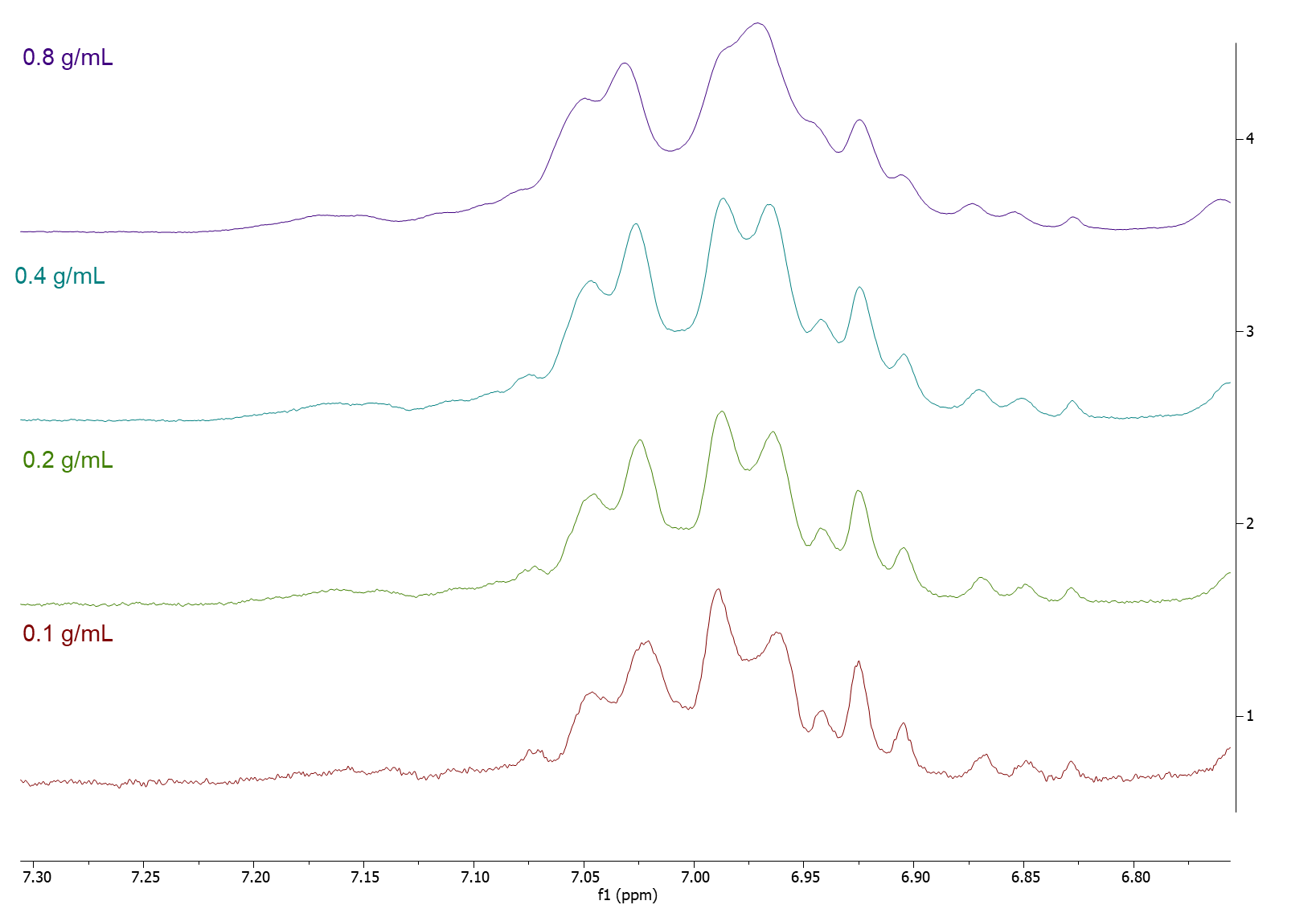


**Figure S9**: Zoomed-in version of the urea moiety of **Figure S7**, showing peak widening and a deep-field shift with decreasing degree of concentration, indicating a decrease of intramolecular interactions (hydrogen bonding).


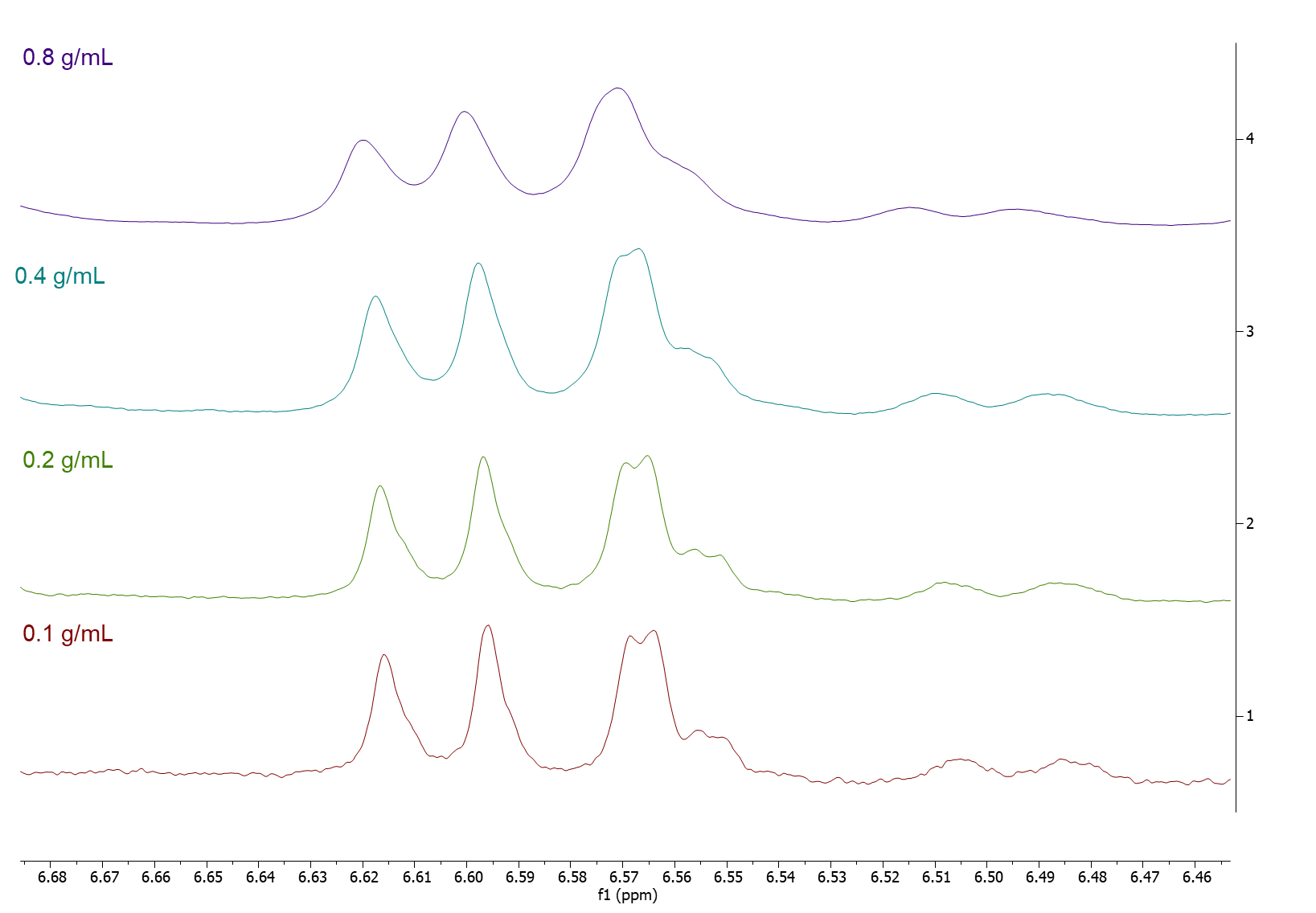


**Figure S10**: Zoomed-in version of the phenyl protons of **Figure S7**, showing peak widening and a deep-field shift with decreasing degree of concentration, indicating a decrease of intramolecular interactions (π-π-stacking).

## **Tensile-Testing**

**Figure S11:** Stress/strain curves of **Mel_1_**_.0_ to **Mel_5_**_.0_.


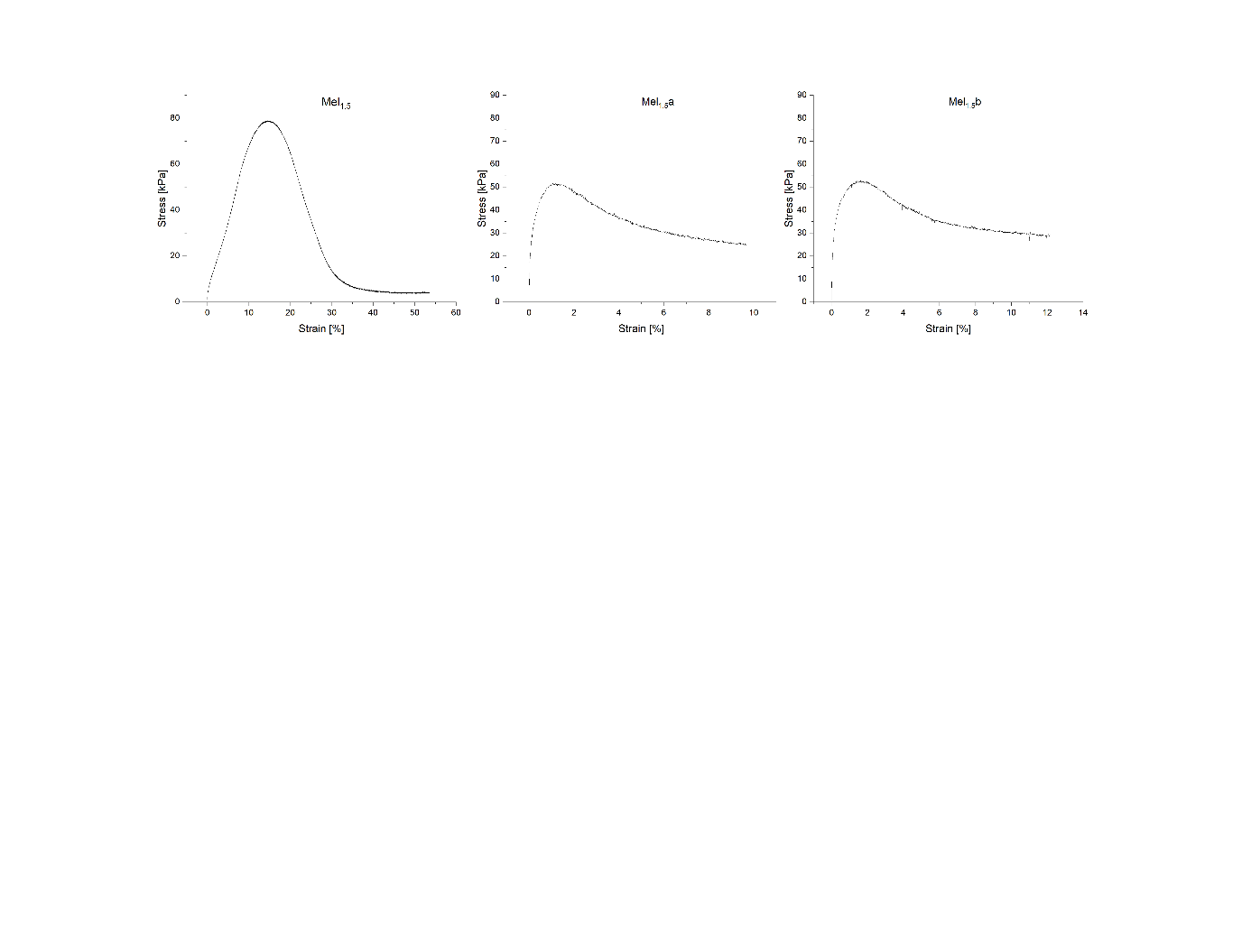


**Figure S12:** Stress/strain curves of **Mel_1_**_._**_5_**, **Mel_1.5_**a and **Mel_1.5_**b.

## **Removability**

**Video S1:** Removal of tensile strength of cast **Mel_1_**_._**_5_** via addition of EtOH.
